# The role of PD-1/PD-L1 in overshooting osteoclastogenesis in periprosthetic joint infections

**DOI:** 10.1038/s42003-025-08143-3

**Published:** 2025-05-22

**Authors:** Yi Ren, Denise Jahn, Stefanie Donner, Clemens Gwinner, Weijie Du, Dimitrios L. Wagner, Serafeim Tsitsilonis, Carsten Perka, Georg Duda, Arne Kienzle

**Affiliations:** 1 Center for Musculoskeletal Surgery, Clinic for Orthopedics, Charité – Universitätsmedizin Berlin, Corporate Member of Freie Universität Berlin, Humboldt-Universität zu Berlin, and Berlin Institute of Health, Berlin, Germany; 2 Julius Wolff Institute and Center for Musculoskeletal Surgery, Charité – Universitätsmedizin Berlin, Corporate Member of Freie Universität Berlin, Humboldt-Universität zu Berlin, and Berlin Institute of Health, Berlin, Germany; 3 BIH Center for Regenerative Therapies (BCRT), Berlin Institute of Health (BIH) at Charité - Universitätsmedizin Berlin, Charitéplatz 1, Berlin, Germany; 4 Berlin Center for Advanced Therapies (BeCAT), Charité - Universitätsmedizin Berlin, corporate member of Freie Universität Berlin, Humboldt-Universität zu Berlin, and Berlin Institute of Health (BIH), Augustenburger Platz 1, Berlin, Germany; 5 Institute of Transfusion Medicine, Charité - Universitätsmedizin Berlin, corporate member of Freie Universität Berlin, Humboldt-Universität zu Berlin, and Berlin Institute of Health (BIH), Berlin, Germany; 6 Institute of Medical Immunology, Charité - Universitätsmedizin Berlin, corporate member of Freie Universität Berlin, Humboldt-Universität zu Berlin, and Berlin Institute of Health (BIH), Berlin, Germany; 7 Berlin Institute of Health at Charité - Universitätsmedizin Berlin, BIH Biomedical Innovation Academy, BIH Charité Clinician Scientist Program, Berlin, Germany

**Keywords:** Translational research, Target identification

## Abstract

Periprosthetic joint infection (PJI) is a critical complication following arthroplasty, leading to increased prosthesis failure rates post-treatment. This study explores the role of PD-1/PD-L1 signaling in osteoclastogenesis associated with PJI. Peripheral blood, bone, and bone marrow of 65 patients (20 primary osteoarthritis, 21 PJI septic explantation, 24 PJI prosthesis reimplantation) were analyzed for their bone microstructure and cell composition. Immunocytochemistry, RT-qPCR, flow cytometry, bone resorption assay, ELISA, and RNA sequencing were performed to investigate the effects of PD-1 stimulation and blockade on osteoclast formation. PD-1 positive monocytes and sPD-L1 levels were elevated in PJI. Stimulation with PD-L1 enhanced osteoclastogenesis, while PD-1 inhibitor nivolumab reversed these effects. Impact of PD-1 and nivolumab was significantly more pronounced in PJI compared to the control. Our study suggests PD-1/PD-L1 signaling plays a significant role in PJI-related osteoclastogenesis. These findings highlight the potential of PD-1 inhibitors as a novel approach to manage this challenging clinical condition.

## Abstract (web summary)

PD-1/PD-L1 signaling drives osteoclastogenesis in PJI. PD-1+ monocytes and sPD-L1 were elevated in PJI, enhancing osteoclast formation. Nivolumab reversed this effect, suggesting PD-1 inhibitors as a potential treatment for PJI-related bone loss.

## Introduction

Periprosthetic joint infection (PJI) is a critical complication following joint arthroplasty that invariably necessitates surgical intervention, often leading to prosthetic failure and mandating complex revision procedures. The primary objective of revision surgery is the meticulous excision of all infected components, encompassing both the prosthesis and the adjacent osseous tissues, to halt the progression of the infection. Following revision surgery for PJI, a significant number of patients experience prosthesis failure due to aseptic loosening, necessitating additional surgical procedures despite successful eradication of the initial infection^1,2^. However, the underlying mechanisms are poorly understood, challenging the prevailing hypothesis that bone stock should recuperate post-operatively^3,4^. Our recent work has demonstrated that affected patients exhibit a pronounced upregulation of inflammatory markers in the bone stock surrounding the prosthesis, coupled with alterations in bone homeostasis—characterized by enhanced bone turnover, increased osteoclast differentiation and activity, and loss of bone mass—suggesting these factors as potential contributors to the increased risk for aseptic loosening^1,5^. These localized alterations in the bone bear resemblance to those observed in osteoporosis^6^.

Current therapeutic strategies for managing bone resorption include bisphosphonates and the RANKL inhibitor denosumab, which are staples in the treatment of osteoporosis. Nevertheless, their potential application in the context of PJI is controversial. Investigations have indicated an association between the use of bone resorption inhibitors and an increased risks of osteonecrosis and heightened bacterial load, thus constraining their utility in PJI cases^7,8^. In contrast, targeting the programmed cell death 1 (PD-1) receptor—an immune checkpoint found on multiple immune cell types including monocytes—has emerged as a promising avenue: Inhibiting PD-1 has already gained clinical acceptance for counteracting immunosuppression in oncology and has been found to limit osteolytic processes in bone metastases^9,10^. However, in contrast to these findings, recent evidence indicated that immune checkpoint inhibitors may be less effective in bone metastases and could be associated with impaired bone metabolism and a subsequent increased fracture risk^11^. The PD-1/PD-L1 axis has been suggested to be implicated in modulating polymorphonuclear and monocytic myeloid-derived suppressor cells (MDSCs) during sepsis-induced immunosuppression, with PD-L1 being more highly expressed in polymorphonuclear MDSCs^12^.

Immune checkpoint inhibitors targeting the PD-1/PD-L1 axis have revolutionized cancer therapy by boosting the immune response against tumors^13^. However, their effects on bone health remain less well understood. Recent discoveries have linked PD-1 to changes in bone dynamics—preclinical models have shown a decrease in osteoclasts leading to osteopetrosis in PD-1 knockout mice^14^, while murine models of tumorigenesis have shown a reduction in osteoclast differentiation within metastatic sites following PD-1 inhibitor therapy^15^. However, Wang et al. found no significant difference in trabecular bone formation between wild-type and PD-1 knockout mice^15^. These discrepancies suggest a complex role for PD-1 signaling in bone metabolism, which warrants further investigation. Unlike bisphosphonates and denosumab, which target bone resorption, PD-1 inhibitors are designed to enhance immune responses and have demonstrated safety in systemic infections, with potential to limit sepsis progression^16,17^. This evidence posits PD-1 blockade as a potentially effective and safe therapeutic modality for PJI.

These cumulative findings pivot the role of PD-1 in PJI into a new focal point, suggesting that its modulation could represent a novel therapeutic frontier for addressing the complex interplay of immune regulation and bone metabolism to improve local bone health and limit post-PJI prosthesis failure. Despite encouraging preclinical data, the direct implications of PD-1 pathways in human PJI remain to be elucidated. Our study aims to fill this gap, exploring the role of PD-1/PD-L1 signaling in osteoclastogenesis within the context of PJI and assessing the therapeutic potential of PD-1 inhibitors to ameliorate the pathological bone turnover in affected patients.

## Materials and methods

### Patients and sample collection

This investigation received approval from the Ethics Committee of Charité University Hospital (reference EA1/110/23) and conformed to the principles of the Declaration of Helsinki. All individuals undergoing staged revision knee arthroplasty due to PJI from January 2023 through November 2023 were considered for inclusion in the study, with informed consent acquired in writing from each participant.

PJI was identified adhering to the criteria set forth by the European Bone and Joint Infection Society (EBJIS) and was ascertained through a standardized protocol^18^. Total knee arthroplasty (TKA) reimplantation was performed in patients without clinical signs of persisting infection, negative infectious parameters (i.e., C-reactive protein [CRP] and white blood-cell count), and at least 6 weeks after implant removal. As comparators, tissue samples from subjects undergoing TKA for primary osteoarthritis served as non-PJI controls. Osteoarthritic patients undergoing primary TKA were selected as controls due to the accessibility of their samples, as it is not feasible to collect bone marrow samples from healthy individuals. While osteoarthritis involves inflammation, these patients provide a practical non-infectious comparison group. Exclusion criteria for this study encompassed any of the following conditions: (1) a pre-existing diagnosis of osteoporosis, (2) TKA due to non-primary osteoarthritis, (3) patients treated with DAIR (debridement, antibiotics and implant retention) procedures or one-stage revision TKA rather than two-stage revision TKA, or (4) infection with HIV.

In this study, peripheral blood, bone marrow, and bone tissue specimens of affected patients were analyzed. In total, 65 specimens were obtained intraoperatively (20 primary osteoarthritis control, 21 PJI septic explantation, and 24 PJI prosthesis reimplantation samples). X-ray microtomography and histological analyses were performed with all samples; cell culture assays were performed with a total of five specimens per group due to limitations in tissue availability.

During surgery, cubic bone samples measuring roughly 3 to 4 cubic millimeters were harvested from the distal femur next to the bone-cement interface. Post-extraction, any adherent soft tissue and sclerotic regions were carefully excised. Bone marrow specimens were intramedullary aspired from the femur using a large syringe. Additionally, we recorded the paraclinical parameters age, gender, ASA score, BMI, causative pathogen, and the Krenn-Morawietz pathological classification of the tissue specimens.

### X-ray microtomography

3-dimensional micro-computer tomography (µCT) scans were performed using a Skyscan 1172 scanner (Bruker, Billerica, MA, USA) at an isotropic voxel size of 10.05 mm, 80 kV, and 124 mA. After acquisition, the raw image datasets were reconstructed at a predefined global threshold range of 0 to 24, determined by the analysis of the gray-level histogram, at 16-bit stacks using NRecon v1.7.4.6 (Skyscan, 2005-11; Bruker microCT 2012-18). The reconstituted bone architecture was analyzed within selected regions of interest (ROI) that encompassed trabecular bone compartments using ImageJ (version 1.53; National Institutes of Health, USA) and 3D Slicer (Slicer Community). ROIs were selected from trabecular bone compartments located approximately 5 mm from the implant interface. In the control group, ROIs were chosen from analogous regions in the distal femur. The ROIs were defined by a length of 1.5 mm and width of 1.5 mm, ensuring consistent anatomical landmarks were used across all samples. Values are reported as a percentage of the control to emphasize variations relative to baseline measurements in patients not affected by PJI, as well as temporal changes at the time of surgery. The reporting of all imaging data adhered to the established guidelines set forth by the American Society for Bone and Mineral Research for tissue imaging documentation^19^.

### Histology

For the preparation of both cryogenic and paraffin-embedded tissue sections, bone specimens were dissected from the distal femur and proximal tibia during surgical procedures and fixed in 4% paraformaldehyde (PFA). For cryosectioning, the specimens were initially incubated in PFA for four hours, subsequently subjected to a sugar gradient to facilitate infiltration, and finally embedded in SCEM medium (Section Lab Co Ltd., Hiroshima, Japan). Transversal sections of 7 µm thickness were produced with a Cryotome (Leica CM3050S, Leica Microsystems, Wetzlar, Germany) and mounted onto microscope slides with cryofilm (Cryofilm type II C, Section Lab Co Ltd., Japan). For the creation of paraffin sections, tissues were fixed in 4% PFA for 48 h at 4 °C, rinsed, and decalcified over four weeks at 37 °C before embedding in paraffin. Sections of 4 µm were cut using a microtome (Leica RM2235, Leica Microsystems).

Histological examination involved tartrate-resistant acid phosphatase (TRAP) staining to visualize osteoclasts. Osteoclasts were imaged using a bright field microscope (Leica DM6B, Leica Microsystems), and analyzed histomorphometrically with ImageJ (version 1.53 u, http://rsbweb.nih.gov/ij/) following the American Society for Bone and Mineral Research guidelines. For immunofluorescence, frozen sections were treated with Tris-Buffered Saline, permeabilized by 0.25% Triton/PBS solution for 10 min, and then blocked in 5% goat serum/5% donkey serum/3% BSA/PBS before overnight incubation with primary antibodies (anti- Cathepsin K (CTSK), 1:400, mouse monoclonal, sc-48353, Santa Cruz, USA; anti-CD68, 1:500, HPA048982, Atlas Antibodies AB, USA). After washing, sections were exposed to secondary antibodies (anti-mouse AF555, 1:400, A32727, Invitrogen; anti-rabbit AF647, 1:400, 406414, Biolegend, Netherlands), mounted with Fluromount-G containing DAPI (Thermo Fisher Scientific, USA), and imaged on a Leica SP5 confocal microscope (Leica) equipped with a Mai Tai HP multiphoton laser (Spectra Physics, USA).

### Monocyte isolation and culture

Blood was drawn pre-surgery using EDTA tubes and processed within 30 min for PBMC isolation via Ficoll® Paque Plus (17-1440-02, Cytiva, USA) density gradient centrifugation. Monocytes were isolated from PBMCs using CD14 magnetic beads (130-050-201, Miltenyi Biotec, Germany) and cultured in alpha-MEM (BE12-169F, Lonza, Switzerland) with 10% FBS (Lot. 42F4484K, Gibco, Thermo Fisher Scientific), 2 mM UltraGlutamine (BE17-605E/U1, Lonza, Switzerland), and 25 mM HEPES (15630080, Thermo Fisher Scientific), plus 25 ng/ml M-CSF (300-25, Peprotech, USA). Media were refreshed bi-daily. On day 4, cultures received additional RANKL (25 ng/ml), PD-1 inhibitor (800 ng/ml; nivolumab; OPDIVO®, USA), and PD-L1 (40 ng/ml; 762506, BioLegend) in various combinations, continuing until day 18 for assays.

### Flow cytometry

PBMCs and bone marrow samples were incubated with an Fc blocking reagent (422302, Biolegend) before antibody staining. Anti-CD33 AF488, 1:50 (11-0338-42, ThermoFisher), anti-CD14 PE-Cyanine7, 1:100 (25-0149-42, ThermoFisher), and anti-PD-1 PE, 1:50 (329906, Biolegend) were used for staining. Additionally, anti-CD45 Brilliant Violet 785 (1:200, 304047, Biolegend) was used for bone marrow staining due to the mixture of hematopoietic and mesenchymal lineage cells.

Stained cells were acquired using an FACSymphony™ A5 Cell Analyzer (BD Biosciences) with a minimum of 10^6 cells. Monocytes were gated for using CD33 and CD14 staining in the PBMC group. In bone marrow samples, CD45-positive cells were gated first to identify the hematopoietic lineage, followed by monocyte identification using CD33 and CD14. Data were analyzed using FlowJo V10.6.2 (Tree Star).

### Cell viability assay

Monocytes were seeded in a 96-well plate and treated with PD-L1 and nivolumab in gradient concentrations. Viability was measured at 24, 48, and 72 h using the CCK-8 assay (CCK-8, AR1160, Boster Biological Technology, USA).

### Phagocytosis assay

Monocytes were incubated with FITC-labeled dextran (0.5 mg/ml, FD40, Sigma-Aldrich, Germany) and assayed for phagocytic activity after 7 and 14 days. Unspecific binding was controlled by incubation at 4 °C, and flow cytometry measured median fluorescence intensity.

### Bone resorption assay

Bone slices prepared from locally obtained bovine femur shafts and disinfected in 70% ethanol were placed in culture wells. Monocytes were seeded and cultured, as described in the section *‘*Monocyte Isolation and Culture. RANKL and M-CSF were added to induce osteoclast differentiation. After 18 days, specimens were fixed with PFA, and cells were removed from bone slices by sonication before staining with toluidine blue (Sigma-Aldrich). Imaging was done with light microscopy (Leica DVM2500) and SEM (Zeiss GeminiSEM 300, Carl Zeiss, Germany).

### Immunofluorescent staining for osteoclasts

Fixed monocytes were stained with TRAP by incubation in Fast Red Violett LB Salt (F3381, Sigma-Aldrich)/Naphthol AS-MX phosphate (N4875, Sigma-Aldrich) buffer for 30 min. Cells were further stained with Phalloidin (1:1000, ab176753, Abcam, UK), and DAPI (1:1000, MBD0015, Sigma-Aldrich) to identify osteoclasts, defined by TRAP-positivity and multiple nuclei. Images were captured with Axio Observer 7 (Carl Zeiss, Germany) and analyzed using Image J and Zeiss ZEN lite software (Carl Zeiss).

### Gene expression analysis

Cells were homogenized for total RNA isolation using RNasy mini Kit (Qiagen). RNA was reverse transcribed to complementary DNA using RevertAid First Strand cDNA Synthesis Kit (ThermoFisher). RT-qPCR was conducted using Power SYBR Green PCR Master Mix (Sigma Aldrich) with Quantstudio 5 (Applied Biosystems, Thermo Fisher Scientific). Expression levels were normalized to *GAPDH* and compared to control samples from primary TKA surgery patients. Target genes included *PDCD1*, *NFATC1*, *CTSK*, *MMP9*, *ACP5*, *CRLR*, *RAMP1*, *CALCR*, *RANK* and *RANKL* (Supplemental Table 1), and expression level of each gene was normalized to housekeeping gene *Glyceraldehyde-3-phosphate dehydrogenase* (*GAPDH*).

### Protein assays

Plasma sPD-L1 and sPD-1 levels were measured using LEGENDplex Human Immune Checkpoint Panel 1 kit (Cat.740867, Biolegend). TRAP and CTSK concentrations were quantified in cell lysates and medium by colorimetric detection assays (Acid Phosphatase Assay Kit Fluorometric, ab83370, Abcam; ELISA kit for CTSK, SEA267Hu, Cloud-Clone, USA). Both intracellular and extracellular activity were tested. Plasma TRAP activity was also determined post-deactivation of erythrocyte-derived TRAP enzymes by incubation at 37 °C for 1 h^20^.

### RNA sequencing

Monocytes were collected from PJI patients at the time of explantation. These monocytes were cultured for 14 days with osteoclastic stimulation, after which RNA was extracted and sequenced. RNA integrity was confirmed (RNA integrity number: 8.2–9.8) using an Agilent TapeStation system (Agilent Technologies, USA), and sequencing was performed on NextSeq® 2000 (Illumina, USA) with a read length of 72 bp after library preparation. Sequencing libraries were generated using the NEBNext Ultra II RNALibrary Prep Kit (New England Biolabs, USA). Using a molecular barcode, samples were demultiplexed (bcl convert 3.8.4) to fastq data and quality controlled (FastQC, version 0.12.1). Trimmomatic (version 0.38) was used for adapter trimming and read filtering. The resulting reads were aligned to the reference genome (GRCh38.108) using Hisat2 (version 2.2.1). The aligned reads were sorted using SAMtools (version 1.9). Reads mapped to each feature were counted using HTSeq (version 0.11.2). Differential expressed genes (DEGs) were computed using DESeq2 R package (version 1.38.1). The resulting gene set was uploaded into the ingenuity pathway analysis (IPA, QIAGEN, USA) program to identify pivotal canonical pathways and disease features. Heatmap, volcano plot, and figures were generated using R (version 4.3.0, R Foundation for Statistical Computing, Vienna, Austria).

### Statistical analysis

All data were collected and recorded in Microsoft® Excel® 2016 (version 2111 Build 16.0.14701.20240, Microsoft, USA). Where applicable, data were presented as mean or median and analyzed for significance using Friedman’s Test or two-way ANOVA. All statistical analyses and plots were performed using R software (R Development Core Team; version: 3.6.3). P-values less than 0.05 were considered statistically significant. Biological replicates were defined as independent patient samples. Technical replicates were included for RT-qPCR, ELISA, and bone resorption assays. Experiments were conducted on at least three independent biological samples.

### Reporting summary

Further information on research design is available in the Nature Portfolio Reporting Summary linked to this article.

## Results

### Demographics

In our cohort, 53.8% of the patients were male, with a mean age of 69.1 years and an average BMI of 29.5 kg/m^2^. 95% had more than one comorbidity and the median ASA score was 2. No significant differences in clinical or paraclinical characteristics were found between the control group and PJI patients at both explantation and reimplantation stages (Table 1). Infectious agents in PJI patients were identified through standard microbiological culture and PCR techniques, performed by our hospital’s microbiology department following established clinical protocols for pathogen detection. At the time of prosthesis explantation, *Staphylococcus* species, primarily *S. aureus*, *S. epidermidis*, and *S. hominis*, were the most prevalent pathogens, accounting for 57.5% of PJI cases.

**Table 1:** Patient Characteristics

	Control			PJI - Explantation				PJI - Reimplantation			
Descriptive	Count	Mean	Range	Count	Mean	Range	P	Count	Mean	Range	P
All patients	20			21				24			
Age [years]		69.0	54.0-80.0		69.7	53.0-91.0	0.211		68.7	49.0-85.0	0.466
Male	8			12			0.252	14			0.226
Female	12			9				10			
BMI		28.6	22.8-36.7		30.1	24.7-43.6	0.130		29.6	20.4-43.6	0.237
ASA Score											
1	3			0			0.352	0			0.187
2	13			16				16			
3	4			5				7			
4	0			0				1			

### µCT Analysis of Bone Specimens

**Fig. 1:**
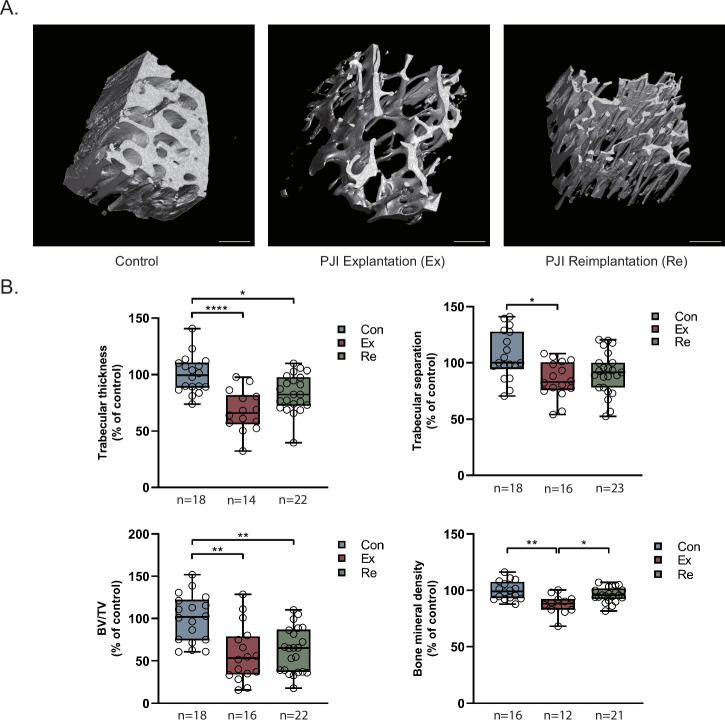
µCT scans of intra-operatively obtained femoral bone specimens. **A** Representative three-dimensional reconstruction of bone microstructure. Scale bar: 500 µm. **B** Average trabecular thickness, separation, BV/TV, and bone mineral density. *P < 0.05; **P < 0.01; ***P < 0.001; ****P < 0.0001. Con, control group; Ex, PJI explantation surgery; Re, PJI reimplantation surgery; BV/TV, bone volume/total volume.

µCT imaging, as previously discussed in our related work^5^, revealed compromised bone health in PJI patients, depicting a more porous and irregular structure in PJI patients (Fig. 1A). Consistent with our previous research, the trabecular architecture in PJI patients was markedly altered, with reduced trabecular thickness at explantation (67.9% of control, *p* < 0.001) and reimplantation (85.2% of control, *p* = 0.088) and a lowered bone volume to total volume ratio (BV/TV) at explantation (58.3% of control, *p* = 0.001) and reimplantation (62.6% of control, *p* = 0.003; Fig. 1B). Trabecular separation was modestly reduced at the time of explantation (79.8% of the control group, p = 0.020). This contrasts with our previous findings, where trabecular separation was increased at explantation, possibly reflecting differences in patient cohorts. Additionally, bone mineral density was negatively impacted during explantation, albeit showing recovery at reimplantation (Fig. 1B). Despite a trend toward improved values at reimplantation, the relative measurements indicate that bone homeostasis remains significantly compromised, failing to fully recover by the time of reimplantation.

### Osteoclast and Macrophage Prevalence

**Fig. 2:**
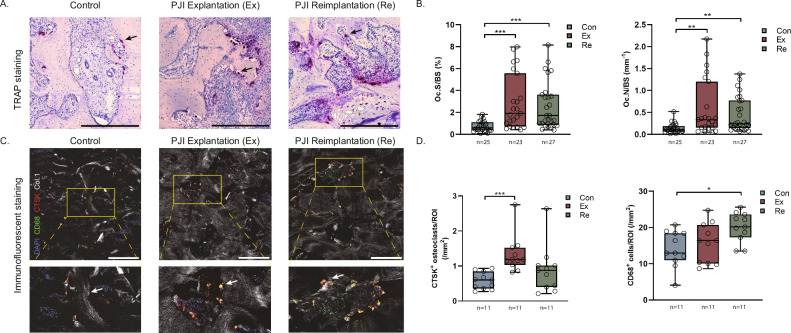
PJI-induced local osteoclastogenesis. **A** TRAP staining for osteoclasts. Scale bar: 500 µm. **B** Histomorphometric quantification of osteoclast-related parameters. Oc.N/BS: Number of osteoclasts per bone surface; Oc.S/BS: Osteoclast surface per bone surface. **C** Distribution of CTSK and CD68 positive cells in the trabecular bone structure. Scale bar: 500 µm. **D** Quantification of CTSK positive (osteoclasts) and CD68 positive (macrophages) cells.

Enhanced osteoclast formation in PJI patients was evident in TRAP staining at both explantation (Oc.N/BS: 0.107 vs 0.334 cells/mm, p = 0.001; Oc.S/BS: 0.585% vs 1.92%, p < 0.001) and reimplantation (Oc.N/BS: 0.107 vs 0.238 cells/mm, p = 0.001; Oc.S/BS: 0.585% vs 1.72%, p < 0.001; Fig. 2A, B). CTSK staining confirmed osteoclasts as a relevant cell population linked to changes in the bone tissue in PJI (Fig. 2C, D), with more CTSK-positive cells observed at explantation compared to control. Conversely, CD68+ macrophage distribution was not linked to these changes and remained consistent at explantation but increased at reimplantation (Fig. 2C, D). In contrast, in bone marrow, CD68^+^ macrophages were found to be elevated at both explantation and reimplantation (Supplemental Fig. 1A-B). Blood TRAP activity, indicative of bone resorption, was elevated in explantation (2.841 vs 5.444 mU/mL, p = 0.004) but normalized during reimplantation (Supplemental Fig. 1C). These findings highlight a distinct increase in osteoclast activity and bone resorption during explantation in PJI patients, which partially resolves by the time of reimplantation.

### PD-1/PD-L1 in Monocytes

**Fig. 3:**
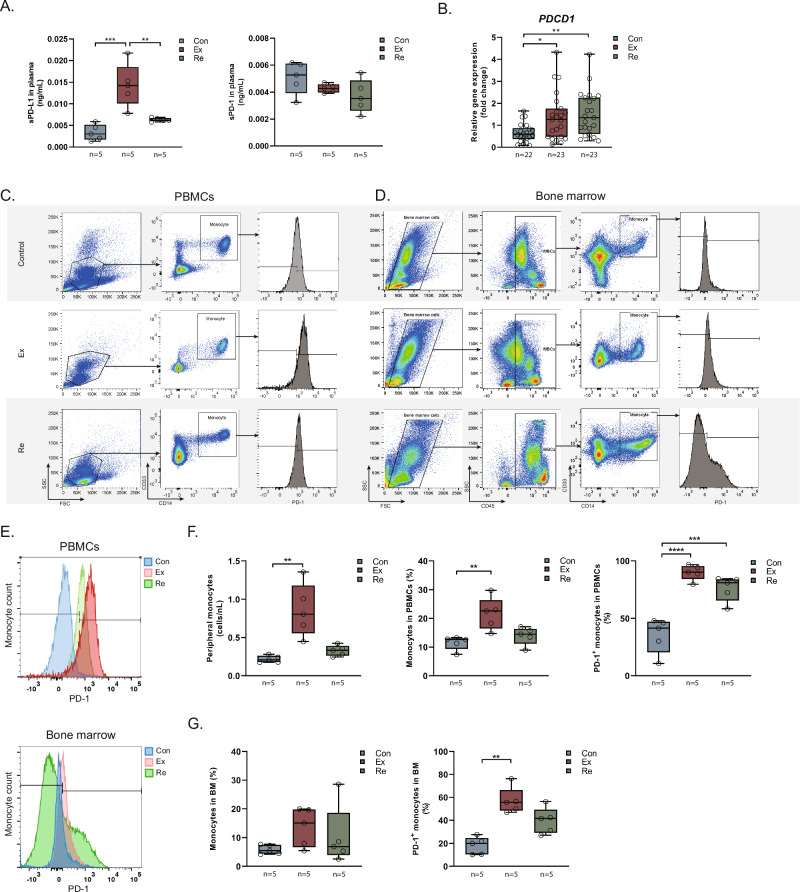
Characteristics of monocytes and PD-1/PD-L1 signaling in PJI. **A** Concentration of sPD-L1 and sPD-1 in serum as determined by ELISA. **B** Gene expression of *PDCD1* in femoral bone. **C**, **E** Flow cytometry of PBMCs: prevalence of monocytes and PD-1 expression. Monocytes were gated using CD33 and CD14 markers. **D**, **E** Flow cytometry using bone marrow specimens: prevalence of monocytes and PD-1 expression. CD45-positive cells were gated first to exclude mesenchymal cells, followed by gating with CD33 and CD14 to identify monocytes. **F**, **G** Quantification of monocyte occurrence and PD-1 expression in PBMCs and bone marrow.

Compared to controls, sPD-L1 levels in plasma were significantly higher in PJI patients at explantation (0.003 vs 0.014 ng/mL, p = 0.001) and were reduced compared to at explantation but elevated compared to the control at reimplantation (0.003 vs 0.006, *p* = 0.325; Fig. 3A). In contrast, sPD-1 levels did not differ significantly among groups (Fig. 3A). Expression of *PDCD1*, the coding gene of PD1, was significantly elevated in femoral bone specimens of patients with PJI at explantation and reimplantation (Fig. 3B). Flow cytometry further revealed both increased PD-1 positive monocytes and elevated cellular PD-1 expression in PJI in peripheral blood (control: 48.0% vs explantation: 96.8%, p < 0.001; vs reimplantation: 84.5%, p < 0.001; Fig. 3C, E, F) and bone marrow specimens (control: 20.1% vs explantation: 55.7%, p = 0.003; vs reimplantation: 41.6%, p = 0.198; Fig. 3D, E, G). This pronounced upregulation of PD-1 expression and sPD-L1 levels in PJI patients indicates their potential role in the local and systemic immune response to infection.

### Effect of PD-L1 and PD-1 neutralization on osteoclast differentiation

**Fig. 4:**
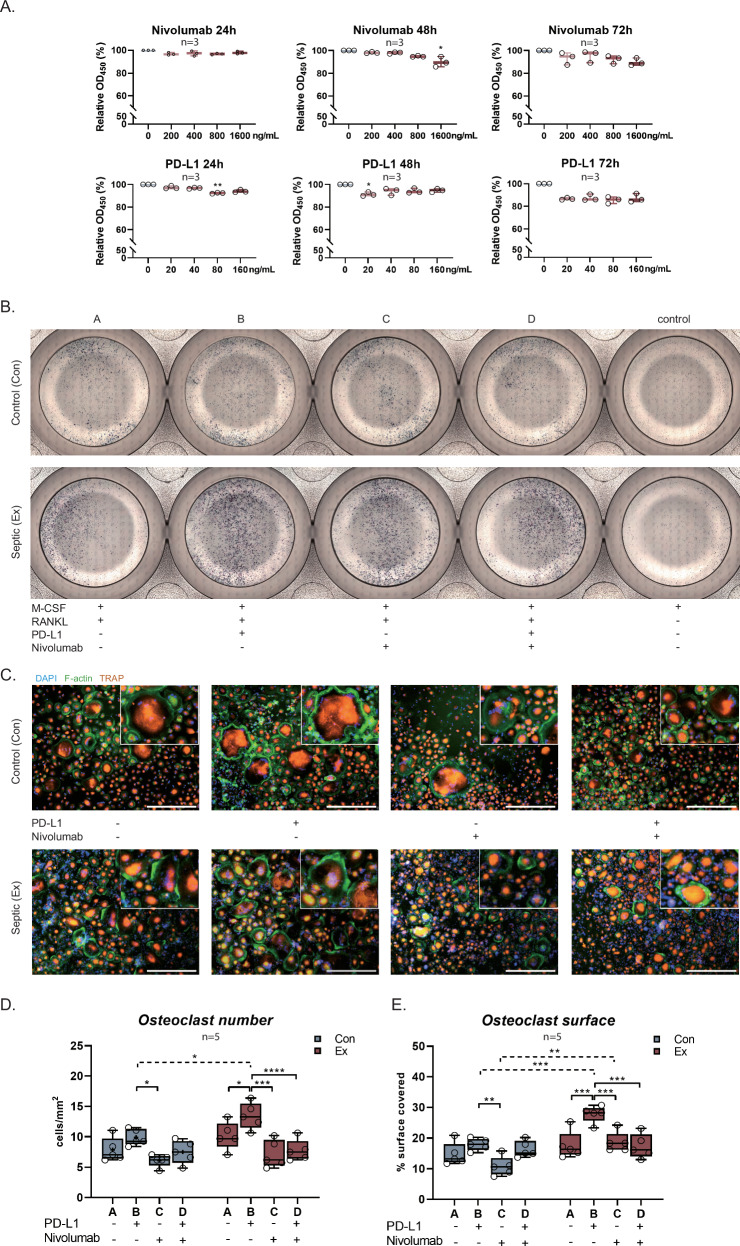
In vitro osteoclast stimulation indicates the role of PD-1 signaling. **A** CCK-8 test for gradient concentration of PD-L1 and PD-1 inhibitor nivolumab for monocytes at time point 24 h, 48 h, and 72 h. **B** Representative overview of osteoclast generation with PD-L1 and/or nivolumab from monocytes of patients at explantation surgery in bright field. **C** Representative immunofluorescent images of monocytic osteoclast differentiation. Scale bar: 200 µm. **D**, **E**) Histomorphometric quantification of osteoclast number and % surface cover.

CCK-8 assay demonstrated minimal in vitro impact on cell viability in monocytes of PD-L1 and anti-PD-1 antibody nivolumab after treatment of up to 72 h (Fig. 4A). Osteoclast formation and surface area covered by osteoclasts, assessed macroscopically and using high-resolution immunohistochemistry staining, were significantly increased by PD-L1 treatment in PJI-derived monocytes (+36.4% osteoclasts, p = 0.044; +72.2% osteoclast surface, p < 0.001), with nivolumab effectively inhibiting this increase (-43.3% osteoclasts, p < 0.001; -42.8% osteoclast surface, p < 0.001; Fig. 4B-E). Additionally, after treatment with PD-L1, both osteoclast number and surface were significantly higher in PJI (osteoclasts: 13.263 vs 9.284 cells/mm2, p = 0.021; osteoclast surface: 17.9% vs 28.2%, p < 0.001, Fig. 4B-E).

### Osteoclast Activation and Function

**Fig. 5:**
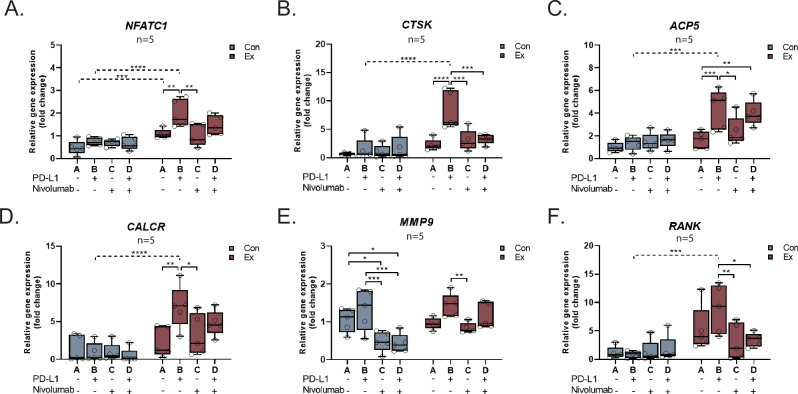
PD-1/PD-L1 signaling and osteoclast gene expression. **A**-**F** Quantification of gene expression in osteoclasts as assessed by RT-qPCR.

RT-qPCR analysis was conducted to evaluate the expression of osteoclast function-related markers in cultured osteoclasts. *NFATC1* expression was upregulated with stimulation with PD-L1 (1.663-fold, p = 0.002), reduced to 0.801-fold (p = 0.999 compared to control) following nivolumab treatment and at 1.301-fold (p = 0.625 compared to control) following PD-L1 and nivolumab co-treatment in PJI-derived cell cultures (Fig. 5A). Similar patterns with distinct variations between the PD-L1 and nivolumab groups were observed for *CTSK*, *ACP5*, and *CALCR* (Fig. 5B-D). While *MMP9* and *RANK* expression did not significantly increase with PD-L1 treatment, nivolumab significantly reduced expression of both markers compared with PD-L1 treatment (Fig. 5E, F). Intriguingly, gene expression of all assessed proteins beside *MMP9* were not affected by neither PD-L1 nor nivolumab in the control group, while a marked elevation was noted in the PJI group after PD-L1 treatment compared to controls (Fig. 5E). Of note, expression of *PDCD1* was not affected by PD-L1 or nivolumab in both the control and PJI group (Supplemental Fig. 3).

**Fig. 6:**
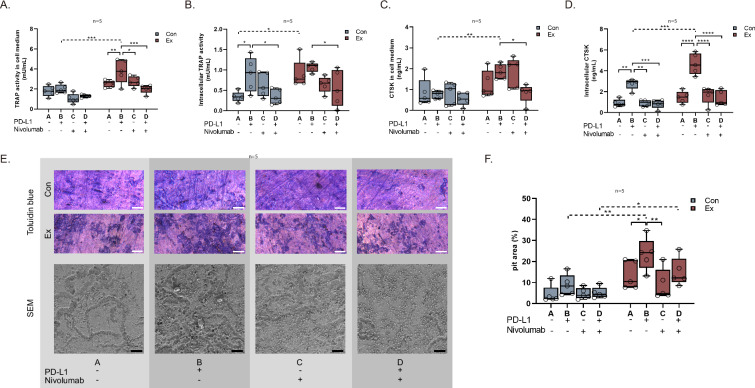
PD-1/PD-L1 signaling and osteoclast function. **A**-**D** TRAP activity and CTSK concentration in cell medium and intracellularly. **E** Toluidine blue staining of pit formation assay and SEM imaging of bone slices following in vitro osteoclast culture. Scale bar for pit assay: 500 µm, for SEM: 100 µm. **F** Quantification of pit formation assay.

Extracellular TRAP activity, indicative of osteoclast activity, was elevated in PJI compared to the control group, especially following PD-L1 treatment (3.781 vs 1.995 mU/mL, p < 0.001; Fig. 6A). Treatment with nivolumab led to a significant decrease in extracellular TRAP activity compared to stimulation with PD-L1 (3.781 vs 1.976 mU/mL, p < 0.001). Intracellular TRAP activity, while elevated in PJI, was less affected compared to extracellular activity, suggesting long-term PD-L1 influence in PJI enhances both intra- and extracellular TRAP activities (Fig. 6B). Further, intracellular TRAP activity decreased with combined PD-L1 and nivolumab treatment in both the control and PJI group (Fig. 6B). Similar trends were seen with CTSK, with levels elevated in PJI and modulated by PD-L1 and nivolumab treatments (Fig. 6C, D). These findings were confirmed by CTSK immunostaining (Supplemental Fig. 4A-B). Of note, intracellular CTSK concentrations exhibited a PD-L1 dose-dependent pattern (Supplemental Fig. 4C).

Pit formation assay demonstrated overall increased osteoclast bone matrix resorption in PJI compared to the control (Fig. 6E). Treatment with PD-L1 further increased osteoclast resorptive activity (8.86% in control vs 23.5% in PJI, p = 0.001), an effect partially mitigated by PD-1 inhibition in the PJI group (23.5% after PD-L1 treatment vs 8.96% after nivolumab treatment, p = 0.002; vs 15.0% after combined PD-L1 and nivolumab treatment, p = 0.106; Fig. 6F).

Phagocytic activity was analyzed to assess a potential impact on bacterial clearance in PJI. In vitro treatment with nivolumab did not negatively impact the phagocytic activity of monocytes (Supplemental Fig. 2). However, PD-L1 reduced FITC-dextran phagocytosis (-29.8%, p = 0.003), an effect that was significantly mitigated by concurrent nivolumab treatment (+44.9% vs PD-L1 treatment, p = 0.001; Supplemental Fig. 2C).

PD-L1 stimulation enhanced osteoclast function and activity in PJI, as evidenced by increased expression of osteoclast-related markers, extracellular TRAP activity, and bone resorption, while nivolumab effectively mitigates these effects without compromising phagocytic activity.

### RNA Sequencing and Pathway Analysis

**Fig. 7:**
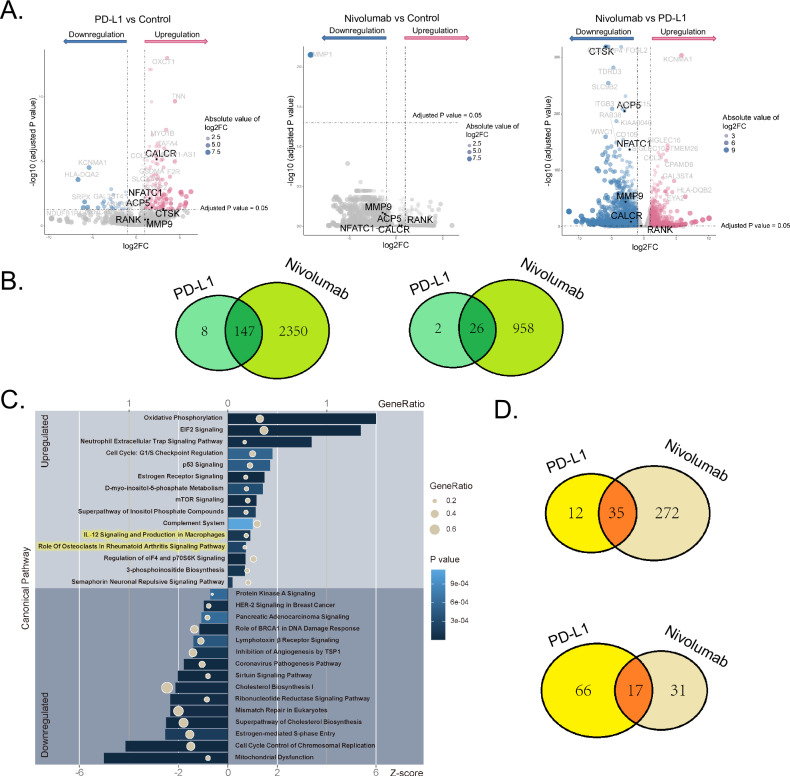
RNA sequencing and bioinformatic analysis. **A** Gene expression fold change in osteoclasts following different treatments. **B** Gene expression overlap of upregulated (left) and downregulated (right) genes in the dataset of control vs PD-L1 treatment and nivolumab vs PD-L1 treatment. **C** Top 15 upregulated and downregulated canonical pathways in osteoclast differentiation. **D** Canonical pathways overlap of upregulated (left) and downregulated (right) pathways in the dataset of control vs PD-L1 treatment and nivolumab vs PD-L1 treatment.

RNA sequencing analysis identified 183 differentially expressed genes (DEGs) in osteoclasts derived from monocytes of PJI patients, stimulated for 14 days with PD-L1. Among these, 155 genes were upregulated, and 28 were downregulated (Supplemental Fig. 5A). Osteoclast-function related gene markers *NFATC1*, *CTSK*, *ACP5*, and *CALCR* were found to be upregulated after PD-L1 treatment. In contrast, *MMP9*, *CRLR*, and *RAMP1* showed no significant expression changes (Fig. 7A, left panel). In contrast, after treatment with nivolumab *MMP1* was the only downregulated gene compared to the control group (Fig. 7A, middle panel). To confirm these alterations in gene expression in PD-1 signaling, sequencing data of the control, nivolumab, and PD-L1 group were compared (Fig. 7A, right panel). Comparing the nivolumab and the PD-L1 group, a total of 2497 genes were recognized as upregulated, while 984 were downregulated after stimulation with nivolumab. Among them, several osteoclast function-related genes were downregulated, including *NFATC1*, *CTSK*, *MMP9*, *ACP5*, and *CALCR*. A comparative analysis of the group analysis (control vs PD-L1 treatment and nivolumab vs PD-L1 treatment) identified 147 common genes that were upregulated and 26 common genes that were downregulated after PD-L1 treatment (Fig. 7B; Supplemental Table 2).

IPA tool revealed key pathways affected by PD-L1 treatment (Fig. 7C). Two upregulated pathways (Z-score > 0) with significant relevance to our cell culture model were identified: “role of osteoclasts in rheumatoid arthritis signaling” and “IL-12 signaling and production in macrophages”. Additionally, other pathways, including p53, mTOR, and p70S6K signaling, exhibited upregulation after PD-L1 induced osteoclastogenesis. Investigating the connection between our gene set and disease models revealed that “bone density” was negatively and “osteopetrosis” positively correlated with our gene expression profile after stimulation with PD-L1 (Supplemental Fig. 5B). A comparative analysis of the group analysis found 35 common pathways that were upregulated and 17 common pathways that were downregulated after PD-L1 treatment compared to both control and nivolumab treatment (Fig. 7D; Supplemental Table 3).

RNA sequencing and pathway analysis revealed that PD-L1 stimulation significantly altered gene expression and activated pathways linked to osteoclastogenesis and inflammatory signaling in PJI, while nivolumab counteracted these changes suggesting a regulatory potential in modulating osteoclast function.

## Discussion

In this study, we explored the role of PD-1/PD-L1 in PJI and its association with altered bone homeostasis in humans, a factor previously linked to increased implant failure rates post-PJI treatment^5,21^. Our analysis revealed that PD-1/PD-L1 signaling contributes significantly to excessive osteoclastogenesis and enhanced osteoclast function subsequently leading to lastingly impared bone homeostasis. Moreover, our data suggest that inhibiting the PD-1/PD-L1 axis could potentially reverse these detrimental effects. Given the high failure rates following PJI due to aseptic loosening with subsequent need for surgical intervention, our findings underscore the urgent need for innovative treatment strategies targeting this mechanism in affected patients.

In PJI, our study observed an increased presence of PD-1 positive monocytes in peripheral blood and bone marrow, alongside a higher macrophage count in the bone marrow compared to controls, indicating active PD-1/PD-L1 signaling during PJI pathogenesis. Correspondingly, sPD-L1 levels were elevated in PJI. In contrast, sPD-1, which acts as a decoy inhibiting activation of the cellular PD-L1 pathway axis, leading to T cell anergy^22^, was not found to be elevated. Previous research on the role of PD-1 in osteoclast formation has mostly focused on osseous metastases and delivered controversial results: While Nagahama et al. observed an increase in bone mineral density and a decrease in osteoclast numbers in PD-1 knockout mice^14^, Wang et al. reported no rise in osteoclast formation in similar knockout mice without tumors, noting bone structure impairment only in those with tumors (12). Conversely, Greisen et al. found PD-1 and PD-L1 knockout models showed signs of osteoporosis^23^. Our findings demonstrate the significance of PD-1/PD-L1 upregulation in excessive osteoclastogenesis and osteoclast function in humans as well. Of note, we found PD-1/PD-L1 signaling to play a pivotal role in osteoclast differentiation in the presence of RANKL in PJI.

Intriguingly, while PD-L1 and nivolumab exhibited discernible effects on osteoclastogenesis and osteoclast resorptive function in the control group, these phenomena were significantly more pronounced in the PJI cohort. This could be due to the observed lower functional PD-1 expression in monocytes without infection or might be potentially modulated by different PD-1 protein isoforms arising from alternative splicing^24^. These findings suggest nivolumab as a potential preventive treatment against PJI-induced excessive osteoclastogenesis and that its usage in PJI may not adversely affect physiological osteoclast function. Cotreatment with PD-L1 and nivolumab resulted in reduced osteoclast formation compared to PD-L1 alone, indicating this therapeutic approach to be feasible in vivo. Additionally, this data suggests that off-target effects of PD-1 are unlikely to be involved in this mechanism and that instead PD-1 is the primary target of PD-L1 in PJI^25^.

High-throughput sequencing technology revealed osteoclast-related genes and pathways affected by PD-1/PD-L1 signaling, further validating our findings. We identified upregulated pathways promoting osteoclast differentiation and survival, with negative implications for bone health. *MMP1* was the only gene affected by nivolumab treatment compared to the control suggesting a limited effect of nivolumab treatment in the absence of PD-L1. However, group comparison found that only a fraction of the up-/downregulated genes and pathways were common for the PD-L1 group compared to both the control and nivolumab treatment group, underscoring the intricate role of PD-1 signaling in osteoclast functionality. Using IPA, we identified canonical pathways and gene modules pertinent to PJI. Notably, an upregulated pathway related to osteoclast function was identified, which enhances osteoclast differentiation, survival, and bone resorption while reducing apoptosis. This pathway’s upregulation was correlated with two crucial disease modules: “bone density” and “osteopetrosis,” suggesting a deterioration in bone health. These findings align with previously published results on the impact of PJI on the bone metabolism and underscore the need for a deeper understanding of the underlying mechanisms at play^5,21^.

Apart from bone remodeling, osteoclasts can function as antigen-presenting cells involved in cellular immunity^26^. Activation of PD-1 signaling in T cells reduces antibacterial immunity in sepsis, which might be mitigated by a PD-1 inhibitor^27^. The use of PD-1 inhibitors has been successfully shown to prolong survival in animal models and was found to be safe for human use in sepsis in a phase 1b clinical trial^28,29^. Compared to systemic sepsis, PJI is a local infection associated with significant inflammatory upregulation^21^, encouraging discovery of its potential as a novel treatment strategy in PJI. While this further suggests PD-1 inhibitors could be effective in treating PJI-related bone loss, the role of PD-1/PD-L1 in the clearance of PJI remains unknown. Our findings also demonstrate that PD-1/PD-L1 can reduce phagocytotic activity, a process reversible by nivolumab. Similarly, negative effects on phagocytosis induced by ligation of PD-1 were found in a previous cancer study^30^. Up-regulation of the PD-1 receptor on monocytes has been associated with poor bacterial clearance and a decrease in the release of proinflammatory cytokines^31^. In this study, we demonstrated that treatment with PD-1 inhibitor nivolumab did not affect the internalization of xenobiotics. Neither PD-L1 nor nivolumab directly induced apoptosis of monocytes. These findings suggest that anti-PD-L1 treatment may be also safe to use in non-cancerous pathologies^32^, especially when administered locally.

This study also has several limitations, including its in vitro nature, patient heterogeneity, patient sample size, and the need for further elucidation of the underlying mechanisms. Furthermore, the use of osteoarthritic controls may underestimate the inflammatory response in PJI samples, as osteoarthritis also involves local inflammation. While our in vitro data provide evidence supporting the role of PD-1/PD-L1 signaling in osteoclastogenesis associated with PJI, further studies analyzing monocytes derived from PJI patients and controls, as well as in vivo PJI models, will be necessary to fully elucidate the functional significance of this pathway in a clinical setting. Nonetheless, our results open avenues for using nivolumab in PJI treatment to limit overshooting osteoclastogenesis without compromising monocyte functionality.

In this study, we found PD-1/PD-L1 signaling to play a crucial role in overshooting osteoclastogenesis in patients with PJI. We demonstrated that this pathway not only contributes to the excessive formation and enhanced function of osteoclasts in PJI but also impacts monocytic phagocytosis, a process reversible by PD-1 inhibition. Our findings suggest that targeting the PD-1/PD-L1 axis could be a promising therapeutic strategy in managing PJI and addressing the high prosthesis failure rates post-treatment. This research paves the way for further investigations into the molecular mechanisms underpinning PJI and opens avenues for novel, targeted interventions.

## Supplementary information

Supplemental Material

Reporting Summary

## Data Availability

The source data underlying all figures and the RNA sequencing data generated in this study are available at Figshare^33–35^. RNA-seq data are provided in Supplementary Table 1. RNA sequencing data generated in this study have been deposited in the Gene Expression Omnibus (GEO) under accession number GSE295042^36^.
