# Supplementary material for: The role of PD-1/PD-L1 in overshooting osteoclastogenesis in periprosthetic joint infections
Source: Commun Biol. 2025 May 22;8:786. doi: 10.1038/s42003-025-08143-3 (PMC12098725; doi:10.1038/s42003-025-08143-3)
Supplement: Supplementary file 1 — Supplemental Material [file 42003_2025_8143_MOESM1_ESM.pdf]

Supplemental Table 1

Gene symbol	Gene name	Forward primer sequence	Reverse primer sequence
GAPDH	Glyceraldehyde-3-Phosphate Dehydrogenase	CTGCACCACCAACTGCTTAG	ACAGTCTTCTGGGTGGCAGT
PDCD1	Programmed Cell Death 1	GGACAATAGGAGCCAGGCG	CCCATAGTCCACAGAGAACACA
NFATC1	Nuclear Factor Of Activated T Cells 1	GGAGATGGAAGCGAAAACTG	GGGGCTGGTTATCCTCTGAT
CTSK	Cathepsin K	TTGGCAGTGGGATATGGAAT	GCCACAGGCGTTGTTCTTAT
MMP9	Matrix Metallopeptidase 9	TCTTCCCTGGAGACCTGAGA	ATTTCGACTCTCCACGCATC
ACP5	Acid Phosphatase 5	CGTGTGGTCCATAGCCGAG	CCACGCCATTCTCATCTTGC
CRLR	Calcitonin Receptor Like Receptor	GCCTAAGTTGCCAAAGGATTACC	TGAACTGGGACACTTTGCAAC
RAMP1	Receptor Activity Modifying Protein 1	AGGTAGACATGGAGGCCGT	ACCTGTCCACCTCTGCATTG
CALCR	Calcitonin Receptor	CAGCAGTTACCCGCATACCA	GGACAATACTCCAGCCGGTG
RANK	TNF Receptor Superfamily Member 11a	TCCACGGACAAATGCAGACC	GCATCGGATTTCTCTGTCCCA
RANKL	TNF Superfamily Member 11	GGCTGGGCCAGGTTGTC	ATTCTATTAGGATCCATCTGCGCT

Supplemental Table 2: Upregulated and downregulated common genes after PD-L1 stimulation in comparison to both control group and Nivolumab treatment.

Common upregulated genes	Common downregulated genes
LINC02416	*CYP1B1*
SLC45A2	*EPS8L1*
SPACA3	*NCR3LG1*
BEST3	*FAHD2B*
ENSG00000214144	*CFAP61*
RPSAP3	*C3*
COL1A2	*ASPHD2*
ANGPTL1	*HLA-H*
NPPC	*ENTPD1*
CAMK2A	*ENSG00000259238*
TNN	*ENSG00000197320*
AFAP1-AS1	*RPS4XP16*
ENSG00000284999	*LINC02972*
NPHS1	*SIGLEC16*
ENSG00000228566	*CPAMD8*
LINC01091	*PVALB*
JCHAIN	*GLB1L*
UOX	*ANKRD35*
F2R	*GAL3ST4*
KIRREL2	*PSME2P3*
SLC9B2	*CXCL10*
SATB1-AS1	*KCNMA1*
OXCT1	*LRRC4C*
ITGA2	*SRPX*
SH3GL2	*NDUFB1P1*
CTNND2	*HLA-DQA2*
MYO1B	
NYAP2	
PTPRK	
REM1	
TAFA4	
DUSP4	
ENSG00000233397	
FSD2	
DGKI	
ENSG00000225885	
GSDMA	
CTSK	
SEMA3D	
SLC6A17	

SLC22A1	
CLDN14	
COL5A1	
PKNOX2	
CLIC6	
EXT1	
SEMA3E	
PKIA	
ITGB3	
SSC5D	
AKAP6	
DPP4	
FHL2	
ENSG00000253227	
STRIP2	
KIAA0040	
ENSG00000230387	
ZNF462	
SHC4	
ATP1B4	
RUFY4	
RPS16P2	
ZEB1	
FOLR3	
RND1	
LRRC8E	
DYRK3	
NEURL3	
SH3D19	
BAMBI	
GRAP2	
SLC9B1	
DNM1	
SYNM	
TPGS2	
LIPH	
CALCR	
PIP5K1B	
PARVA	
OMG	
ADGRB1	
ADAM19	
ATOH8	
LRP6	

N4BP2	
C12orf75	
ENSG00000291236	
SPRY4	
DMPK	
GLI3	
MAP1A	
STAP2	
EGR1	
MYO1D	
PKIG	
GCNT3	
ENSG00000289070	
UST	
STAMBPL1	
OCIAD2	
DNAJA4	
KIAA1614	
EOGT	
LRG1	
ARHGAP23	
HLA-DRB5	
KIAA1328	
ACP5	
AFAP1	
WSCD1	
THOP1	
GOLM1	
LTBP2	
AKR1C1	
RBFOX2	
SDC1	
XPR1	
GHRL	
PPFIBP1	
NAT14	
PHETA1	
SNAPC1	
NFATC1	
PACSIN3	
RCAN1	
RGS10	
STX1A	
LHFPL6	

ENSG00000273341	
RAB11FIP5	
PLXNA2	
BMAL2	
CBLB	
SMARCA1	
RARG	
VWA5A	
GREB1	
JDP2	
STRADB	
C1orf198	
OAF	
BDH1	
PACC1	
NDFIP2	
ZSWIM4	
ZFTA	
DHTKD1	

Supplemental Table 3: Upregulated and downregulated common pathways after PD-L1 stimulation in comparison to both control group and Nivolumab treatment.

Common upregulated pathways	Common downregulated pathways
3-phosphoinositide Biosynthesis	PTEN Signaling
3-phosphoinositide Degradation	Protein Kinase A Signaling
ABRA Signaling Pathway	Adenosine Nucleotides Degradation II
Cell Cycle: G1/S Checkpoint Regulation	Role of BRCA1 in DNA Damage Response
Colorectal Cancer Metastasis Signaling	Leptin Signaling in Obesity
D-myo-inositol (1,4,5,6)-Tetrakisphosphate Biosynthesis	Th1 Pathway
D-myo-inositol (3,4,5,6)-tetrakisphosphate Biosynthesis	Th2 Pathway
D-myo-inositol-5-phosphate Metabolism	Cyclins and Cell Cycle Regulation
Estrogen Receptor Signaling	LXR/RXR Activation
Ferroptosis Signaling Pathway	BER (Base Excision Repair) Pathway
Gustation Pathway	CLEAR Signaling Pathway
Hepatic Fibrosis Signaling Pathway	Mismatch Repair in Eukaryotes
IL-1 Signaling	Ribonucleotide Reductase Signaling Pathway
Insulin Secretion Signaling Pathway	Estrogen-mediated S-phase Entry
Macrophage Alternative Activation Signaling Pathway	Role of Hypercytokinemia/hyperchemokinemia in the Pathogenesis of Influenza
Neuregulin Signaling	Macrophage Classical Activation Signaling Pathway
Neutrophil Extracellular Trap Signaling Pathway	Cell Cycle Control of Chromosomal Replication
NF-κB Activation by Viruses	
p53 Signaling	
PAK Signaling	
Phagosome Formation	
RAR Activation	
Regulation of Cellular Mechanics by Calpain Protease	
RHOA Signaling	
Role of CHK Proteins in Cell Cycle Checkpoint Control	
Role Of Chondrocytes In Rheumatoid Arthritis Signaling Pathway	
Role of NFAT in Cardiac Hypertrophy	
Role Of Osteoclasts In Rheumatoid Arthritis Signaling Pathway	
Role of p14/p19ARF in Tumor Suppression	
Role of Tissue Factor in Cancer	
S100 Family Signaling Pathway	
Semaphorin Neuronal Repulsive Signaling Pathway	

Senescence Pathway	
Superpathway of Inositol Phosphate Compounds	
WNT/Ca+ pathway	

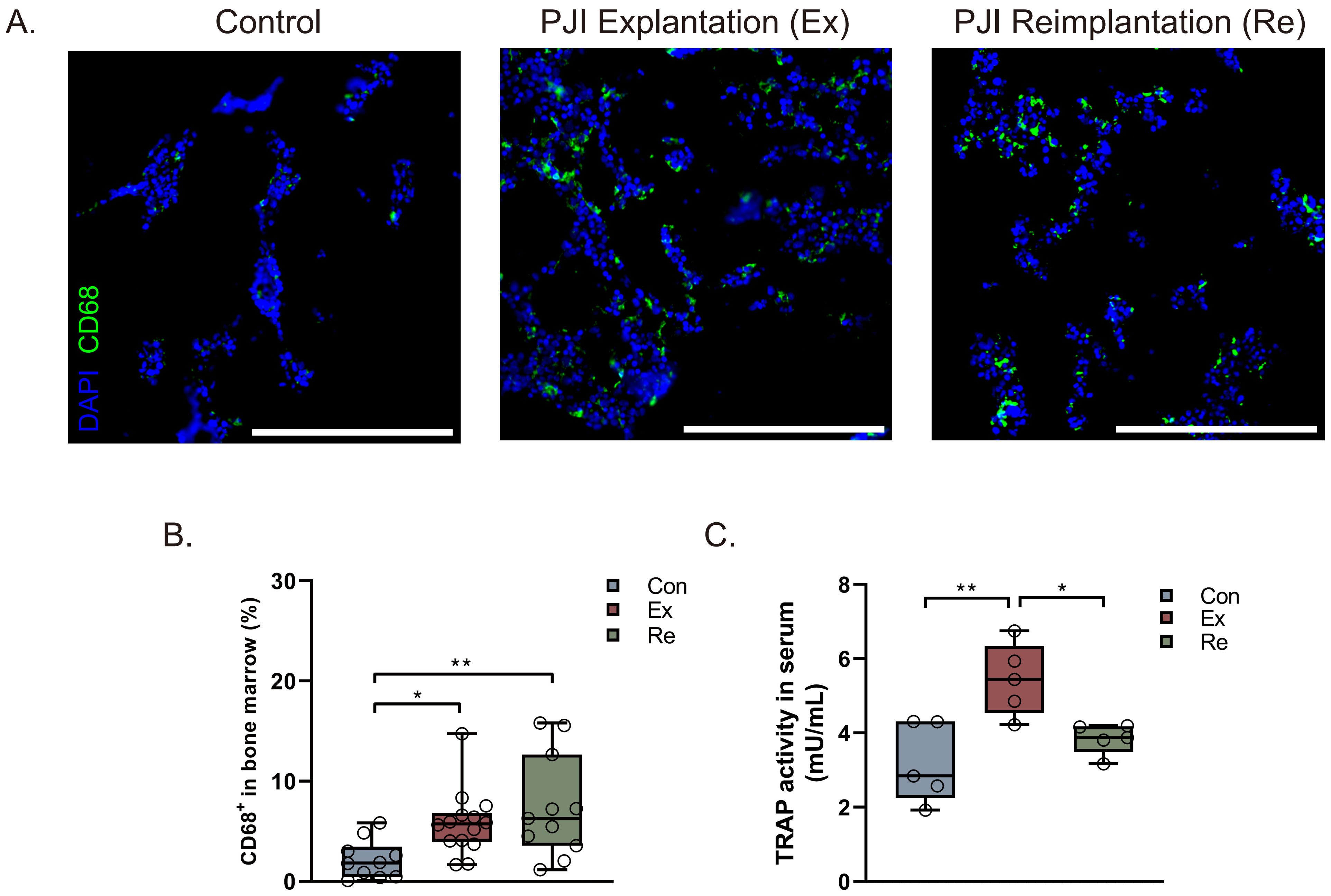
A.
Control
PJI Explantation (Ex)
PJI Reimplantation (Re)
DAPI CD68
B.
CD68+ in bone marrow (%)
30
20
10
0
Con
Ex
Re
*
**
C.
TRAP activity in serum (mU/mL)
8
6
4
2
0
Con
Ex
Re
**
*

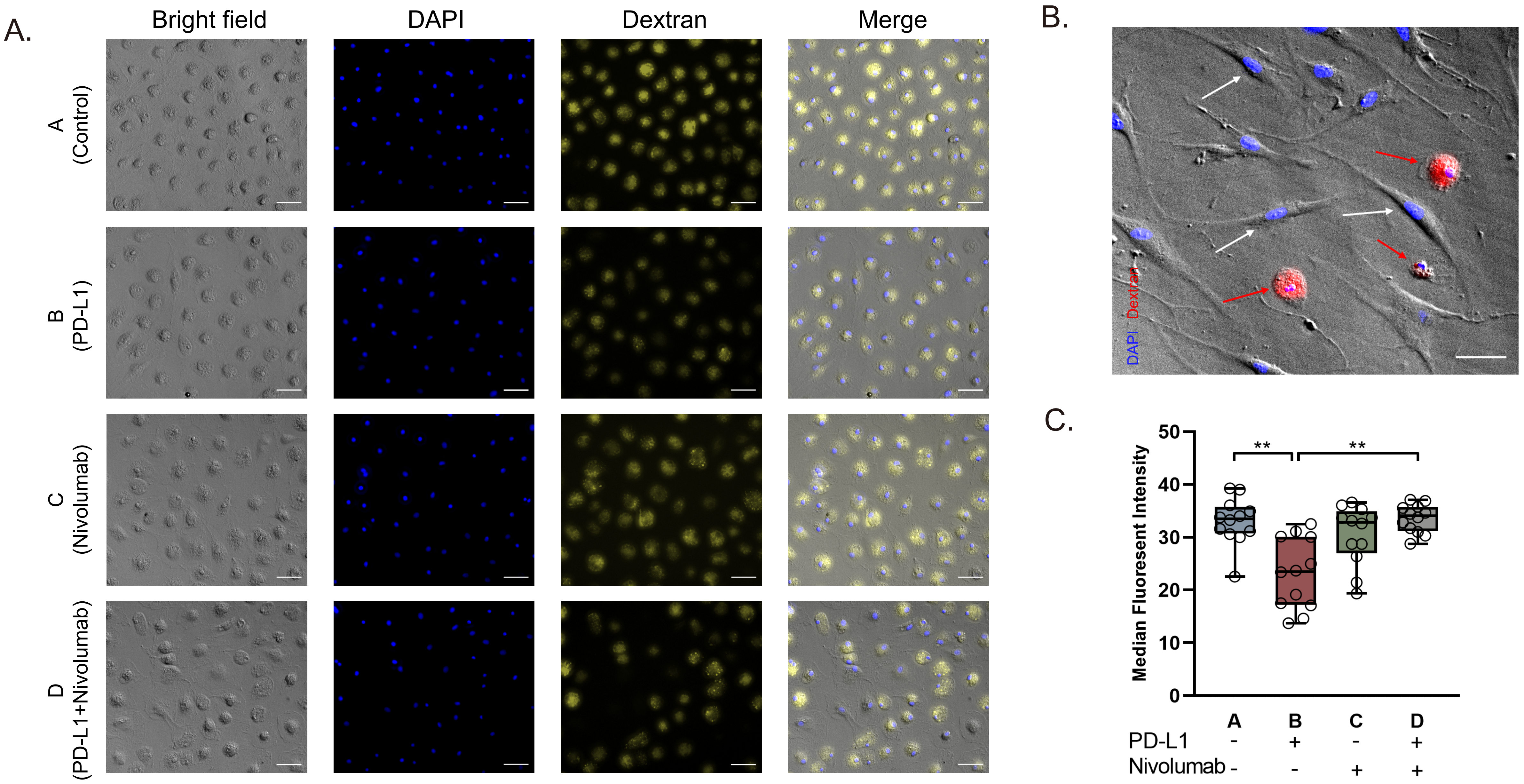
A.
Bright field
DAPI
Dextran
Merge
A (Control)
B (PD-L1)
C (Nivolumab)
D (PD-L1+Nivolumab)
B.
DAPI Dextran
C.
Median Fluoresent Intensity
50
40
30
20
10
0
**
**
A
B
C
D
PD-L1 - + - +
Nivolumab - - + +

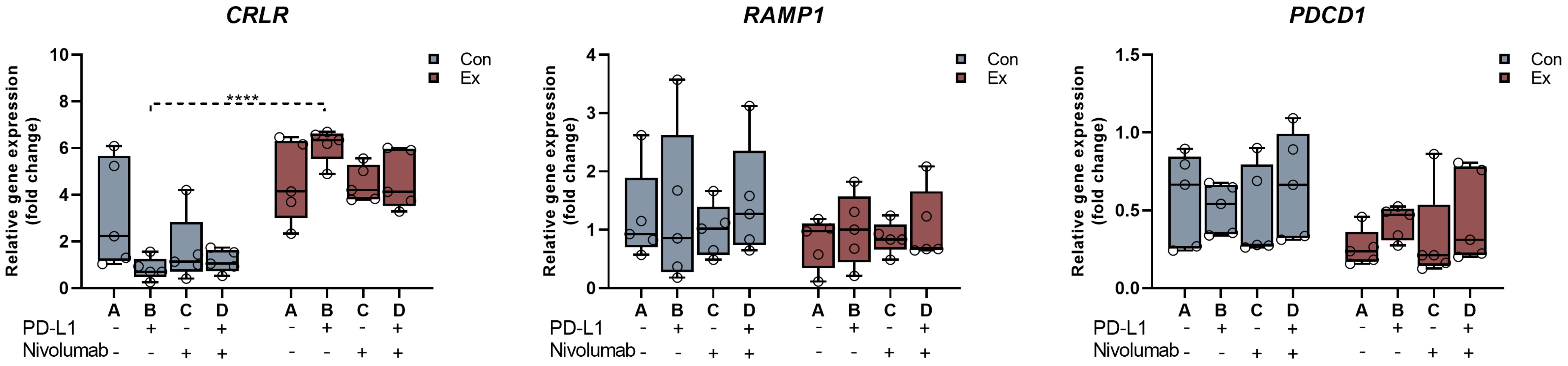

CRLR
RAMP1
PDCD1
Relative gene expression (fold change)
Con
Ex
****
A B C D A B C D
PD-L1 - + - + - + - +
Nivolumab - - + + - - + +

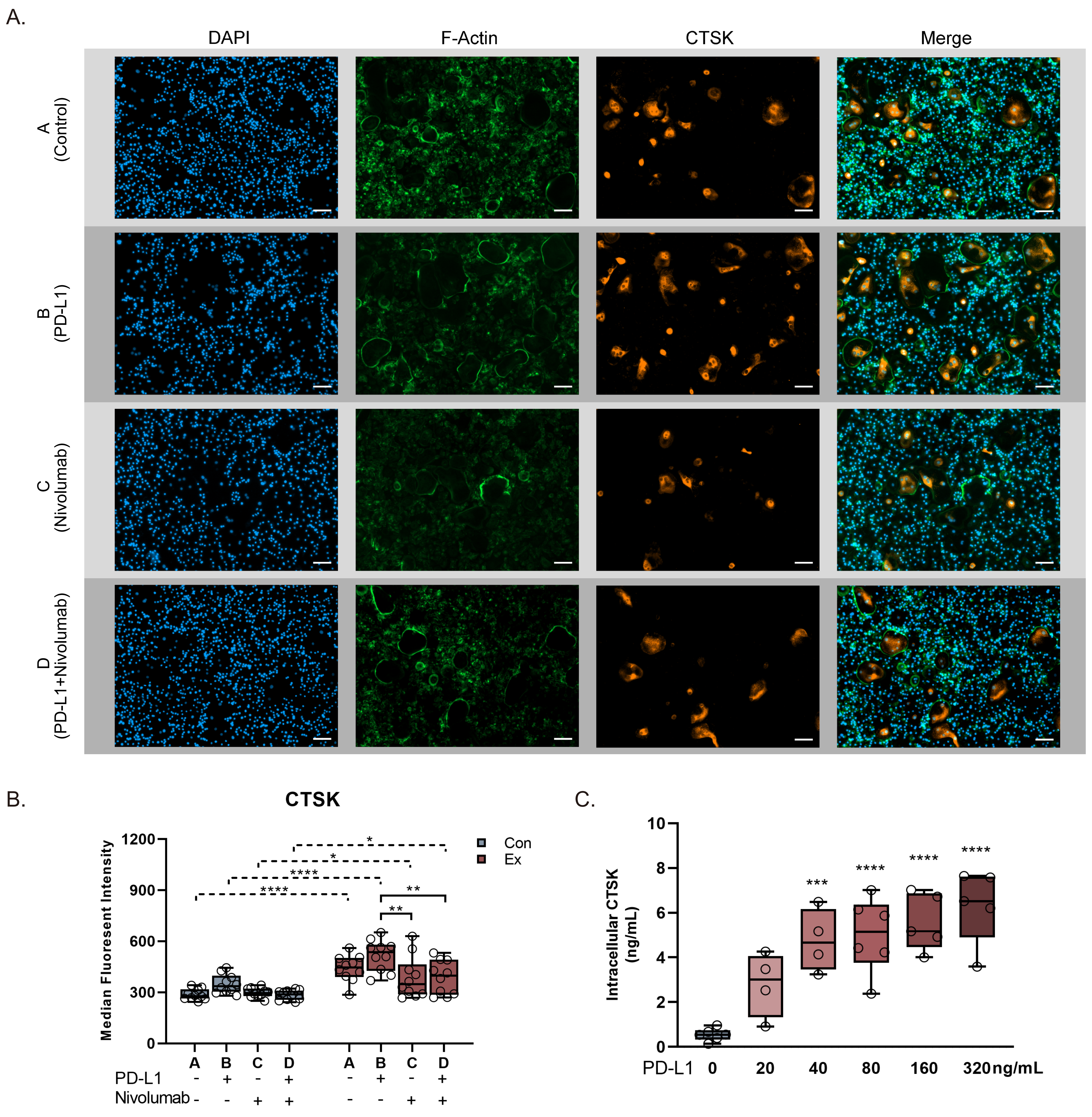
A.
DAPI
F-Actin
CTSK
Merge
A (Control)
B (PD-L1)
C (Nivolumab)
D (PD-L1+Nivolumab)
B.
CTSK
Median Fluoresent Intensity
1200
900
600
300
0
Con
Ex
*
*
****
****
**
**
A B C D A B C D
PD-L1 - + - + - + - +
Nivolumab - - + + - - + +
C.
Intracellular CTSK (ng/mL)
10
8
6
4
2
0
***
****
****
****
PD-L1 0 20 40 80 160 320 ng/mL

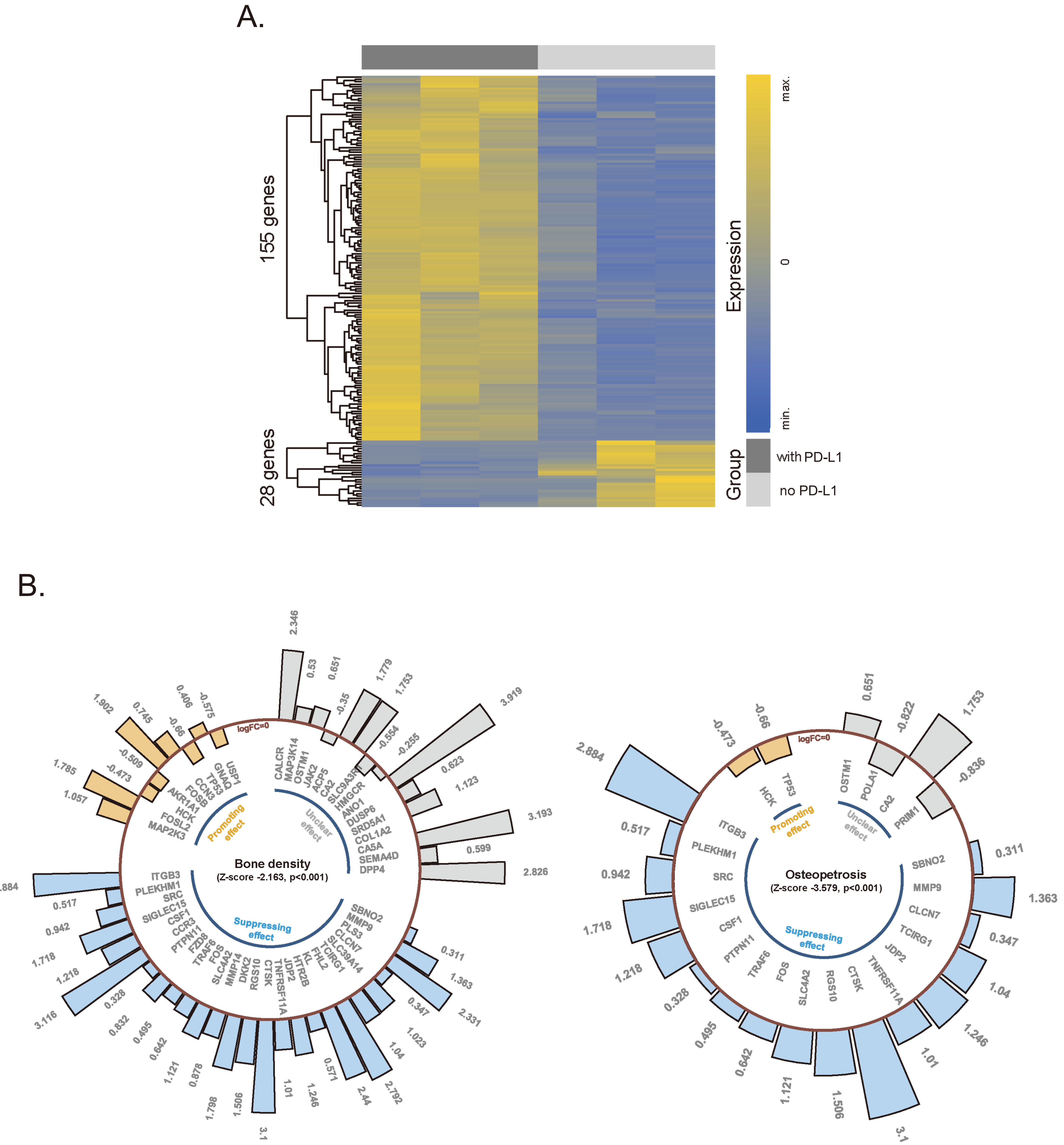
A.
155 genes
28 genes
Expression
max.
0
min.
Group
with PD-L1
no PD-L1
B.
Bone density
(Z-score -2.163, p<0.001)
Promoting effect
Unclear effect
Suppressing effect
logFC=0
Osteopetrosis
(Z-score -3.579, p<0.001)
USP1
GNAQ
TP53
CCN3
FOSB
AKR1A1
HCK
FOSL2
MAP2K3
CALCR
MAP3K14
OSTM1
JAK2
ACP5
CA2
SLC9A3R1
HMGCR
ANO1
DUSP6
SRD5A1
COL1A2
CA5A
SEMA4D
DPP4
ITGB3
PLEKHM1
SRC
SIGLEC15
CSF1
CCR3
PTPN11
FZD8
TRAF6
FOS
SLC4A2
MMP14
DKK2
RGS10
CTSK
TNFRSF11A
JDP2
HTR2B
KL
FHL2
TCIRG1
SLC39A14
CLCN7
PLS3
MMP9
SBNO2
POLA1
PRIM1

Supplement figure 1: (A) Representative images of bone marrow specimens showing distribution of macrophages (CD68 positive cells). Scale bar: 200 µm. (B) Quantification of macrophages in bone marrow. (C) TRAP activity test in plasma among three groups.

Supplement figure 2: (A, B) Representative fluorescent imaging of monocytes after treatment with FITC-labeled dextran at day 7. White arrows, BMSCs; red arrows, monocytes. (C) Median fluorescent intensity of FITC in monocytes acquired in cell culture plate at day 7. BMSCs, bone marrow mesenchymal stem cells.

Supplement figure 3: Quantification of expression of *RAMP1*, *CRLR,* and *PDCD1* in osteoclasts by RT-qPCR indicate osteoclast function.

Supplement figure 4: (A, B) Immune cytochemistry of CTSK in cultured osteoclasts from monocytes in patients at explantation surgery and quantification. Scale bar: 100 µm. (C) Quantification of intracellular CTSK of osteoclasts stimulated with gradient concentrations of PD-L1.

Supplement figure 5: (A) Heatmap plot demonstrating distinct gene expression patterns between osteoclasts stimulated with PD-L1 and without stimulation. Yellow and blue indicates relative up- and downregulation, respectively. (B) Two downregulated disease modules consisting of a list of contributing genes, including those with promoting effect, suppressing effect and unclear effect. IPA, ingenuity pathway analysis.
