# Supplementary material for: The role of PD-1/PD-L1 in overshooting osteoclastogenesis in periprosthetic joint infections
Source: Commun Biol. 2025 May 22;8:786. doi: 10.1038/s42003-025-08143-3 (PMC12098725; doi:10.1038/s42003-025-08143-3)
Supplement: Supplementary file 2 — Reporting Summary [file 42003_2025_8143_MOESM2_ESM.pdf]

nature portfolio

Corresponding author(s): *Double-anonymous peer review submissions: write DAPR and your manuscript number here instead of author names.*

Last updated by author(s): *YYYY-MM-DD*

Reporting Summary

Nature Portfolio wishes to improve the reproducibility of the work that we publish. This form provides structure for consistency and transparency in reporting. For further information on Nature Portfolio policies, see our Editorial Policies and the Editorial Policy Checklist.

Statistics

For all statistical analyses, confirm that the following items are present in the figure legend, table legend, main text, or Methods section.

n/a	Confirmed	
☐	☒	The exact sample size (*n*) for each experimental group/condition, given as a discrete number and unit of measurement
☐	☒	A statement on whether measurements were taken from distinct samples or whether the same sample was measured repeatedly
☐	☒	The statistical test(s) used AND whether they are one- or two-sided<br>*Only common tests should be described solely by name; describe more complex techniques in the Methods section.*
☐	☒	A description of all covariates tested
☐	☒	A description of any assumptions or corrections, such as tests of normality and adjustment for multiple comparisons
☐	☒	A full description of the statistical parameters including central tendency (e.g. means) or other basic estimates (e.g. regression coefficient) AND variation (e.g. standard deviation) or associated estimates of uncertainty (e.g. confidence intervals)
☐	☒	For null hypothesis testing, the test statistic (e.g. *F*, *t*, *r*) with confidence intervals, effect sizes, degrees of freedom and *P* value noted<br>*Give P values as exact values whenever suitable.*
☒	☐	For Bayesian analysis, information on the choice of priors and Markov chain Monte Carlo settings
☐	☒	For hierarchical and complex designs, identification of the appropriate level for tests and full reporting of outcomes
☐	☒	Estimates of effect sizes (e.g. Cohen's *d*, Pearson's *r*), indicating how they were calculated

Our web collection on statistics for biologists contains articles on many of the points above.

Software and code

Policy information about availability of computer code

Data collection	All data were collected and recorded in Microsoft® Excel® 2016 (version 2111 Build 16.0.14701.20240, Microsoft, USA).
Data analysis	All statistical analyses and plots were performed using R software (R Development Core Team; version: 3.6.3).

For manuscripts utilizing custom algorithms or software that are central to the research but not yet described in published literature, software must be made available to editors and reviewers. We strongly encourage code deposition in a community repository (e.g. GitHub). See the Nature Portfolio guidelines for submitting code & software for further information.

Data

Policy information about availability of data

All manuscripts must include a data availability statement. This statement should provide the following information, where applicable:

- Accession codes, unique identifiers, or web links for publicly available datasets
- A description of any restrictions on data availability
- For clinical datasets or third party data, please ensure that the statement adheres to our policy

Provide your data availability statement here.

Research involving human participants, their data, or biological material

Policy information about studies with human participants or human data. See also policy information about sex, gender (identity/presentation), and sexual orientation and race, ethnicity and racism.

Reporting on sex and gender	No reporting on gender. Sex: self-reported by patients
Reporting on race, ethnicity, or other socially relevant groupings	No reporting
Population characteristics	In our cohort, 53.8% of the patients were male, with a mean age of 69.1 years and an average BMI of 29.5 kg/m2. 95% had more than one comorbidity and the median ASA score was 2. No significant differences in clinical or paraclinical characteristics were found between the control group and PJI patients at both explantation and reimplantation stages.
Recruitment	All individuals undergoing staged revision knee arthroplasty due to PJI from January 2023 through November 2023 were considered for inclusion in the study, with informed consent acquired in writing from each participant.
Ethics oversight	This investigation received approval from the Ethics Committee of Charité University Hospital (reference EA1/110/23) and conformed to the principles of the Declaration of Helsinki.

Note that full information on the approval of the study protocol must also be provided in the manuscript.

Field-specific reporting

Please select the one below that is the best fit for your research. If you are not sure, read the appropriate sections before making your selection.

☒ Life sciences ☐ Behavioural & social sciences ☐ Ecological, evolutionary & environmental sciences

For a reference copy of the document with all sections, see nature.com/documents/nr-reporting-summary-flat.pdf

Life sciences study design

All studies must disclose on these points even when the disclosure is negative.

Sample size	In total, 65 specimens were obtained intraoperatively (20 primary osteoarthritis control, 21 PJI septic explantation, and 24 PJI prosthesis reimplantation samples).
Data exclusions	No data exclusions
Replication	All analysis were performed 3 times per sample to ensure reproducibility.
Randomization	N/A
Blinding	N/A

Reporting for specific materials, systems and methods

We require information from authors about some types of materials, experimental systems and methods used in many studies. Here, indicate whether each material, system or method listed is relevant to your study. If you are not sure if a list item applies to your research, read the appropriate section before selecting a response.

Materials & experimental systems

n/a	Involved in the study	
☐	☒	Antibodies
☒	☐	Eukaryotic cell lines
☒	☐	Palaeontology and archaeology
☒	☐	Animals and other organisms
☒	☐	Clinical data
☒	☐	Dual use research of concern
☒	☐	Plants

Methods

n/a	Involved in the study	
☒	☐	ChIP-seq
☐	☒	Flow cytometry
☒	☐	MRI-based neuroimaging

Antibodies

Antibodies used	anti-Cathepsin K (CTSK), 1:400, mouse monoclonal, sc-48353, Santa Cruz, USA; anti-CD68, 1:500, HPA048982, Atlas Antibodies AB, USA); anti-mouse AF555, 1:400, A32727, Invitrogen; anti-rabbit AF647, 1:400, 406414, Biolegend, Netherlands; Anti-CD33 AF488, 1:50 (11-0338-42, ThermoFisher); anti-CD14 PE-Cyanine7, 1:100 (25-0149-42, ThermoFisher); anti-PD-1 PE, 1:50 (329906, Biolegend); anti-CD45 Brilliant Violet 785 (1:200, 304047, Biolegend)
Validation	All primary antibodies used were validated for human samples and their respective applications per manufacturers' data. Validation details, including species reactivity and application, are provided on the manufacturers' websites.

Plants

Seed stocks	N/A
Novel plant genotypes	N/A
Authentication	N/A

Flow Cytometry

Plots

Confirm that:

☒ The axis labels state the marker and fluorochrome used (e.g. CD4-FITC).

☒ The axis scales are clearly visible. Include numbers along axes only for bottom left plot of group (a 'group' is an analysis of identical markers).

☒ All plots are contour plots with outliers or pseudocolor plots.

☒ A numerical value for number of cells or percentage (with statistics) is provided.

Methodology

Sample preparation	see Manuscript
Instrument	see Manuscript
Software	see Manuscript
Cell population abundance	see Manuscript
Gating strategy	see Manuscript, included in main manuscript

☒ Tick this box to confirm that a figure exemplifying the gating strategy is provided in the Supplementary Information.
